# Supplementary material for: Direct observation of coordinated assembly of individual native centromeric nucleosomes
Source: bioRxiv. 2023 May 17:2023.01.20.524981. Originally published 2023 Jan 21. Preprint. [Version 2] doi: 10.1101/2023.01.20.524981 (PMC9882320; doi:10.1101/2023.01.20.524981)
Supplement: Supplement 7 [file NIHPP2023.01.20.524981v2-supplement-7.pdf]

bioRxiv preprint doi: https://doi.org/10.1101/2023.01.20.524981; this version posted May 17, 2023. The copyright holder for this preprint (which was not certified by peer review) is the author/funder, who has granted bioRxiv a license to display the preprint in perpetuity. It is made available under aCC-BY 4.0 International license.

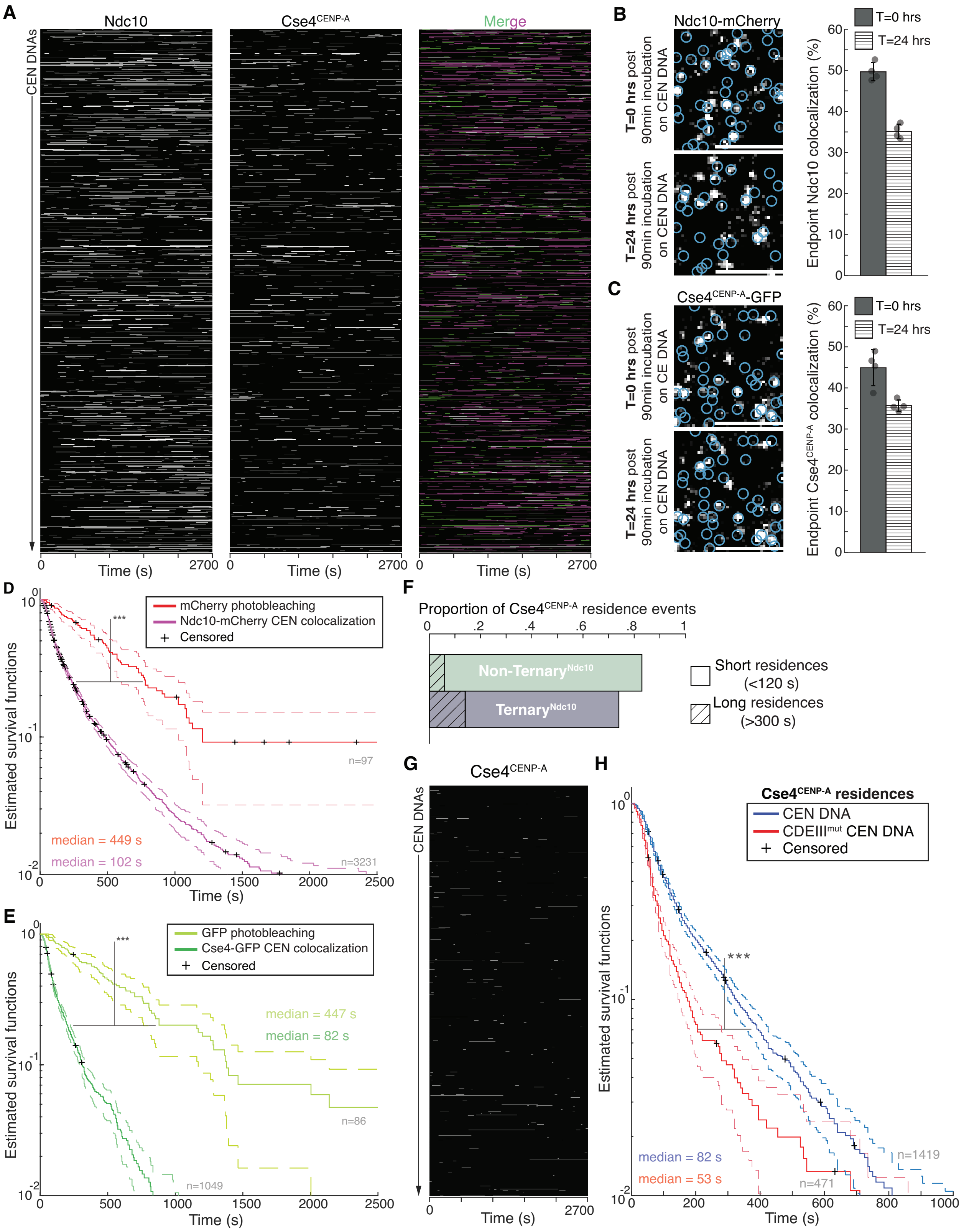

bioRxiv preprint doi: https://doi.org/10.1101/2023.01.20.524981; this version posted May 17, 2023. The copyright holder for this preprint (which was not certified by peer review) is the author/funder, who has granted bioRxiv a license to display the preprint in perpetuity. It is made available under aCC-BY 4.0 International license.

Figure EV1. Ndc10 residence lifetimes are longer than those of Cse4$^{CENP\text{-}A}$, which are not severely limited by photobleaching, while both are extremely stable on CEN DNA once removed from lysate.

A Example plot of Ndc10 and Cse4$^{CENP\text{-}A}$ residence pulses on CEN DNA identified via residence lifetime assays during an entire imaging sequence acquisition. Each row represents one identified CEN DNA with all identified residences shown over entire imaging sequence (2700 s) for Ndc10 (left) and Cse4$^{CENP\text{-}A}$ (center) with merge of Ndc10 (magenta) and Cse4$^{CENP\text{-}A}$ (green) indicating ternary residences (white).

B Example images of TIRFM endpoint colocalization assays. Visualized Ndc10-mCherry on CEN DNA after 90 min incubation and removal of lysate (0 hrs -top panel) or after 24 hrs incubation at RT in imaging buffer (bottom panel) with colocalization shown in relation to identified CEN DNA in blue circles. Scale bars 3 μm. Graph shows quantification of Ndc10 endpoint colocalization on CEN DNA at 0 hrs and 24 hrs (50 $\pm$ 2.2%, 35 $\pm$ 1.7% respectively, avg $\pm$ s.d. n=4 experiments, each examining ~1,000 DNA molecules from different extracts).

C Example images of TIRFM endpoint colocalization assays. Visualized Cse4$^{CENP\text{-}A}$ GFP on CEN DNA after 90 min incubation and removal of lysate (0 hrs -top panel) or after 24 hrs incubation at RT in imaging buffer (bottom panel) with colocalization shown in relation to identified CEN DNA in blue circles. Scale bars 3 μm. Graph shows quantification of Cse4$^{CENP\text{-}A}$ endpoint colocalization on CEN DNA at 0 hrs and 24 hrs (45 $\pm$ 4.4%, 36 $\pm$ 1.4% respectively, avg $\pm$ s.d. n=4 experiments, each examining ~1,000 DNA molecules from different extracts).

D Kaplan-Meier analysis of mCherry photobleaching events (red - median photobleaching lifetimes of 449 s (n=97)) and Ndc10 residences on CEN DNA (magenta - median lifetime of 102 s (n=3231)). There was a significant difference between mCherry photobleaching lifetimes and Ndc10 residence lifetime survival plots (***) on CEN (two-tailed p-value of 0 as determined by log-rank test).

bioRxiv preprint doi: https://doi.org/10.1101/2023.01.20.524981; this version posted May 17, 2023. The copyright holder for this preprint (which was not certified by peer review) is the author/funder, who has granted bioRxiv a license to display the preprint in perpetuity. It is made available under aCC-BY 4.0 International license.

E Kaplan-Meier analysis of GFP photobleaching events (yellow -median photobleaching lifetime of 447 s (n=86)) and Cse4^{CENP-A} residences on CEN DNA (red – median colocalization lifetime of 88 s (n=1054)). There was a significant difference between GFP photobleaching lifetimes and Cse4^{CENP-A} residence lifetime survival plots (***) on CEN DNA (two-tailed p-value of 0 as determined by log-rank test). 95% confidence intervals indicated (dashed lines), right-censored lifetimes (plus icons) were included and unweighted in survival function estimates.

F Quantification of the proportion of short residences (<120 s) and long residences (>300 s) of **Non-Ternary^{Ndc10}** Cse4^{CENP-A} residences (.77 and .06 respectively, n=612 over 3 experiments of ~1000 DNA molecules using different extracts) or **Ternary^{Ndc10}** Cse4^{CENP-A} (.60 and .14 respectively, n=539 over 3 experiments of ~1000 DNA molecules using different extracts).

G Example plot of residences of Cse4^{CENP-A} on CDEIII^{mut} CEN DNA per imaging sequence. Each row represents one identified CEN DNA with all identified residences shown over entire imaging sequence (2700 s) for Cse4^{CENP-A}.

H Cse4^{CENP-A} residence lifetimes on CDEIII^{mut} CEN DNA are reduced. Estimated survival function plots of Kaplan-Meier analysis of residence lifetimes of Cse4^{CENP-A} on CEN DNA (blue – median lifetime of 82 s, n=1419 over 3 experiments of ~1000 DNA molecules using different extracts) and residences on CDEIII^{mut} CEN DNA of Cse4^{CENP-A} (red - of 52 s (n=471 over 3 experiments of ~1000 DNA molecules using different extracts). There was a significant difference (***) between CEN DNA and CDEIII^{mut} CEN DNA lifetime survival plots (two-tailed p-value of 0 as determined by log-rank test). 95% confidence intervals indicated (dashed lines), right-censored lifetimes (plus icons) were included and unweighted in survival function estimates.

bioRxiv preprint doi: https://doi.org/10.1101/2023.01.20.524981; this version posted May 17, 2023. The copyright holder for this preprint (which was not certified by peer review) is the author/funder, who has granted bioRxiv a license to display the preprint in perpetuity. It is made available under aCC-BY 4.0 International license.

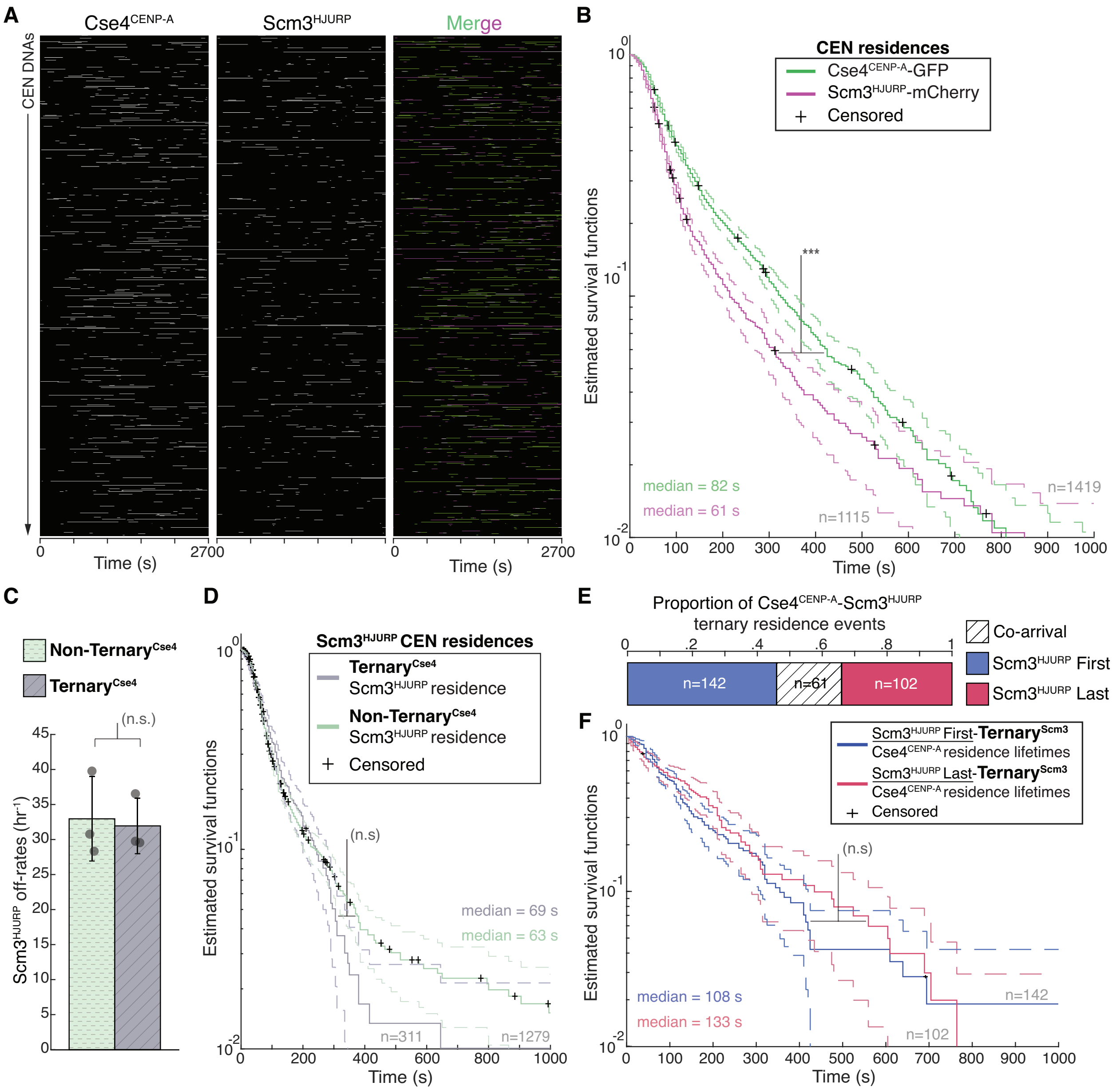

bioRxiv preprint doi: https://doi.org/10.1101/2023.01.20.524981; this version posted May 17, 2023. The copyright holder for this preprint (which was not certified by peer review) is the author/funder, who has granted bioRxiv a license to display the preprint in perpetuity. It is made available under aCC-BY 4.0 International license.

Figure EV2. Scm3^{HJURP} has shorter residence lifetimes than Cse4^{CENP-A} on CEN DNA.

A Example plot of Cse4^{CENP-A} and Scm3^{HJURP} residences on CEN DNA per imaging sequence. Each row represents one identified CEN DNA with all identified residences shown over entire imaging sequence (2700 s) Cse4^{CENP-A} (left) and Scm3^{HJURP} (center) with merge indicating Cse4^{CENP-A} (green), Scm3^{HJURP} (magenta) and ternary residences (white).

B Estimated survival function plots of Kaplan-Meier analysis of Cse4^{CENP-A} residence lifetimes on CEN DNA (green – median lifetime of 82 s, n=1419 over 3 experiments of ~1000 DNA molecules using different extracts), and residence lifetimes of Scm3^{HJURP} on CEN DNA (magenta – median lifetime of 61 s, n=1115 over 3 experiments of ~1000 DNA molecules using different extracts). 95% confidence intervals indicated (dashed lines). Significant difference (***) between Cse4^{CENP-A} and Scm3^{HJURP} residence survival plots (two-tailed p-value of 9.1e-14 as determined by log-rank test), right-censored lifetimes (plus icons) were included and unweighted in survival function estimates.

C Quantification of the estimated off-rates of Scm3^{HJURP} that never formed a ternary residence (**Non-Ternary^{Cse4}**) and of Scm3^{HJURP} after ternary residence with Cse4^{CENP-A} (**Ternary^{Cse4}**) on CEN DNA (114 s $\pm$ 13 s and 111 s $\pm$ 19 s respectively, avg $\pm$ s.d. n=2050 over 3 experiments of ~1000 DNA molecules using different extracts). No significant difference between off-rates (n.s.) with a P-value of .87 as determined by two-tailed unpaired *t*-test.

D Estimated survival function plots of Kaplan-Meier analysis of the lifetimes of **Ternary^{Cse4}** Scm3^{HJURP} residences on CEN DNA (purple – median lifetime of 69 s, n=311 over 3 experiments of ~1000 DNA molecules using different extracts) and **Non-Ternary^{Cse4}** Scm3^{HJURP} residences on CEN DNA (green - of 63 s, n=1279 over 3 experiments of ~1000 DNA molecules using different extracts). No significant difference (n.s.) between **Ternary^{Cse4}** and **Non-Ternary^{Cse4}** survival plots (two-tailed p-value of .75

bioRxiv preprint doi: https://doi.org/10.1101/2023.01.20.524981; this version posted May 17, 2023. The copyright holder for this preprint (which was not certified by peer review) is the author/funder, who has granted bioRxiv a license to display the preprint in perpetuity. It is made available under aCC-BY 4.0 International license.

as determined by log-rank test). 95% confidence intervals indicated (dashed lines), right-censored lifetimes (plus icons) were included and unweighted in survival function estimates.

E Timing of all observed Cse4$^{CENP\text{-}A}$ and Scm3^{HJURP} ternary residence events. The proportion when Scm3^{HJURP} precedes Cse4$^{CENP\text{-}A}$ (Scm3^{HJURP} First) is 0.46, followed by .34 when Cse4$^{CENP\text{-}A}$ precedes Scm3^{HJURP} (Scm3^{HJURP} Last), with a proportion of .20 co-arrival events (Co-arrival - defined as residence initiation within 5 s of each, n=305 over 3 experiments of ~1000 DNA molecules using different extracts).

F Estimated survival function plots of Kaplan-Meier analysis of the lifetimes of Scm3^{HJURP}-First-**Ternary^{Scm3}** Cse4$^{CENP\text{-}A}$ residences on CEN DNA (purple – median lifetime of 108 s, n=142 over 3 experiments of ~1000 DNA molecules using different extracts) and Scm3^{HJURP}-Last-**Ternary^{Scm3}** Cse4$^{CENP\text{-}A}$ residences on CEN DNA (green - of 133 s, n=102 over 3 experiments of ~1000 DNA molecules using different extracts). No significant difference (n.s.) between Scm3^{HJURP}-First and Scm3^{HJURP}-Last survival plots (two-tailed p-value of .59 as determined by log-rank test). 95% confidence intervals indicated (dashed lines), right-censored lifetimes (plus icons) were included and

bioRxiv preprint doi: https://doi.org/10.1101/2023.01.20.524981; this version posted May 17, 2023. The copyright holder for this preprint (which was not certified by peer review) is the author/funder, who has granted bioRxiv a license to display the preprint in perpetuity. It is made available under aCC-BY 4.0 International license.

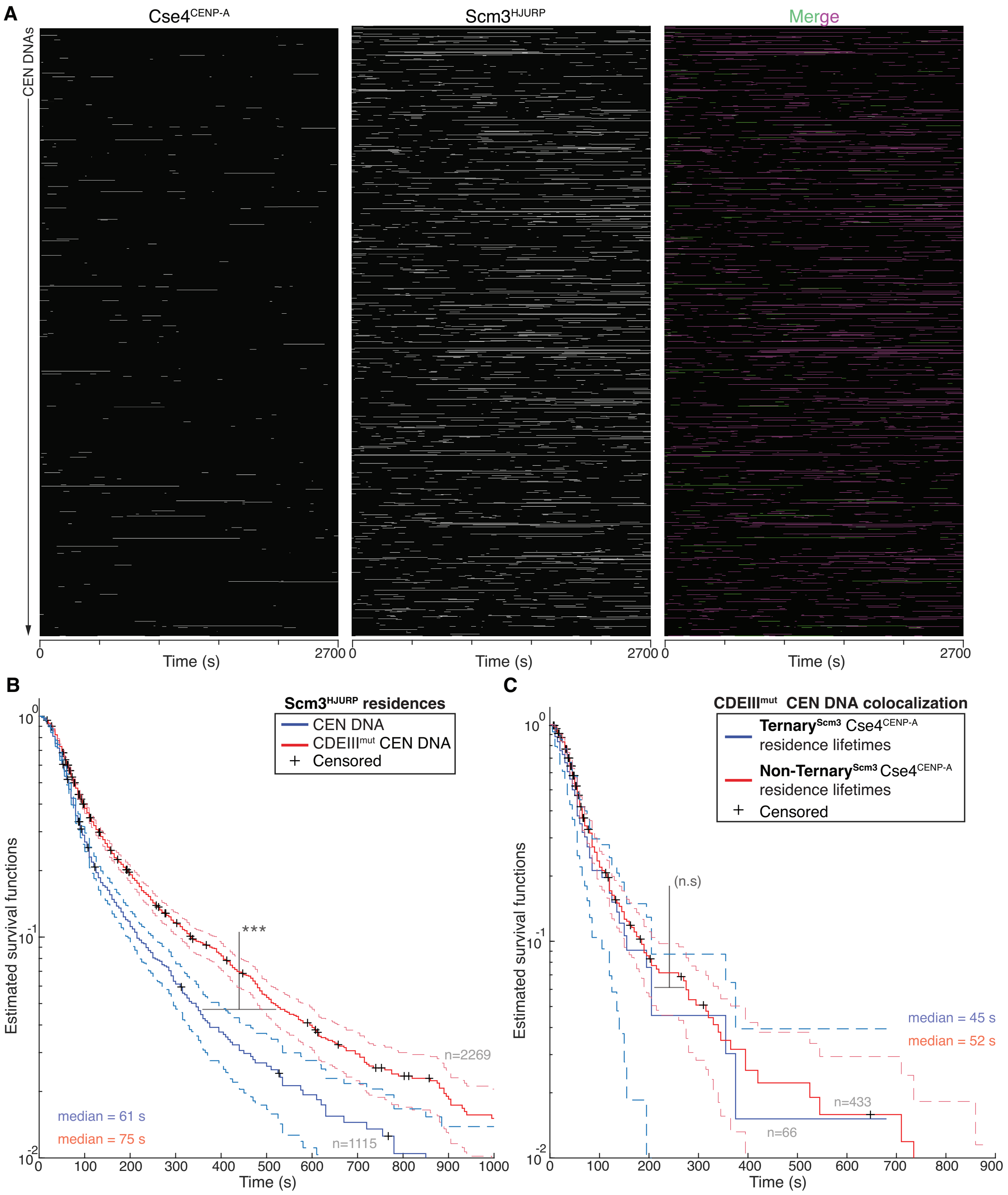

bioRxiv preprint doi: https://doi.org/10.1101/2023.01.20.524981; this version posted May 17, 2023. The copyright holder for this preprint (which was not certified by peer review) is the author/funder, who has granted bioRxiv a license to display the preprint in perpetuity. It is made available under aCC-BY 4.0 International license.

Figure EV3. Cse4$^{CENP\text{-}A}$ interacts transiently with CDEIII^{mut} CEN DNA with or without its chaperone Scm3^{HJURP}.

A Example plot of residences of Cse4$^{CENP\text{-}A}$ and Scm3^{HJURP} on CDEIII^{mut} CEN DNA per imaging sequence. Each row represents one identified CEN DNA with all identified residences shown over entire imaging sequence (2700 s) for Cse4$^{CENP\text{-}A}$ (left) and Scm3^{HJURP} (center) with merge indicating Cse4$^{CENP\text{-}A}$ (green), Scm3^{HJURP} (magenta) and ternary residences (white).

B Scm3^{HJURP} residences are longer on CDEIII^{mut} CEN DNA. Estimated survival function plots of Kaplan-Meier analysis of Scm3^{HJURP} residence lifetimes on CEN DNA (blue - median lifetime of 61 s, n=1115 over 3 experiments of ~1000 DNA molecules using different extracts), and residence lifetimes of Scm3^{HJURP} on CDEIII^{mut} CEN DNA (red - median lifetime of 75 s, n=2269 over 3 experiments of ~1000 DNA molecules using different extracts). There was a significant difference (***) between CEN DNA and CDEIII^{mut} CEN DNA lifetime survival plots (two-tailed p-value of 5.13e-13 as determined by log-rank test). 95% confidence intervals indicated (dashed lines), right-censored lifetimes (plus icons) were included and unweighted in survival function estimates.

C Cse4$^{CENP\text{-}A}$ residence lifetimes are similar without Scm3^{HJURP} a on CDEIII^{mut} CEN DNA. Estimated survival function plots of Kaplan-Meier analysis of ternary residence lifetimes of **Ternary^{Scm3}** Cse4$^{CENP\text{-}A}$ residences on CDEIII^{mut} CEN DNA (blue – median lifetime of 45 s, n=66 over 3 experiments of ~1000 DNA molecules using different extracts) and **Non-Ternary^{Scm3}** Cse4$^{CENP\text{-}A}$ residences on CDEIII^{mut} CEN DNA (red - of 52 s (n=433 over 3 experiments of ~1000 DNA molecules using different extracts). There was no significant difference (n.s.) between **Non-Ternary^{Scm3}** and **Ternary^{Scm3}** survival plots (two-tailed p-value of .27 as determined by log-rank test). 95% confidence intervals indicated (dashed lines), right-censored lifetimes (plus icons) were included and unweighted in survival function estimates.

bioRxiv preprint doi: https://doi.org/10.1101/2023.01.20.524981; this version posted May 17, 2023. The copyright holder for this preprint (which was not certified by peer review) is the author/funder, who has granted bioRxiv a license to display the preprint in perpetuity. It is made available under aCC-BY 4.0 International license.

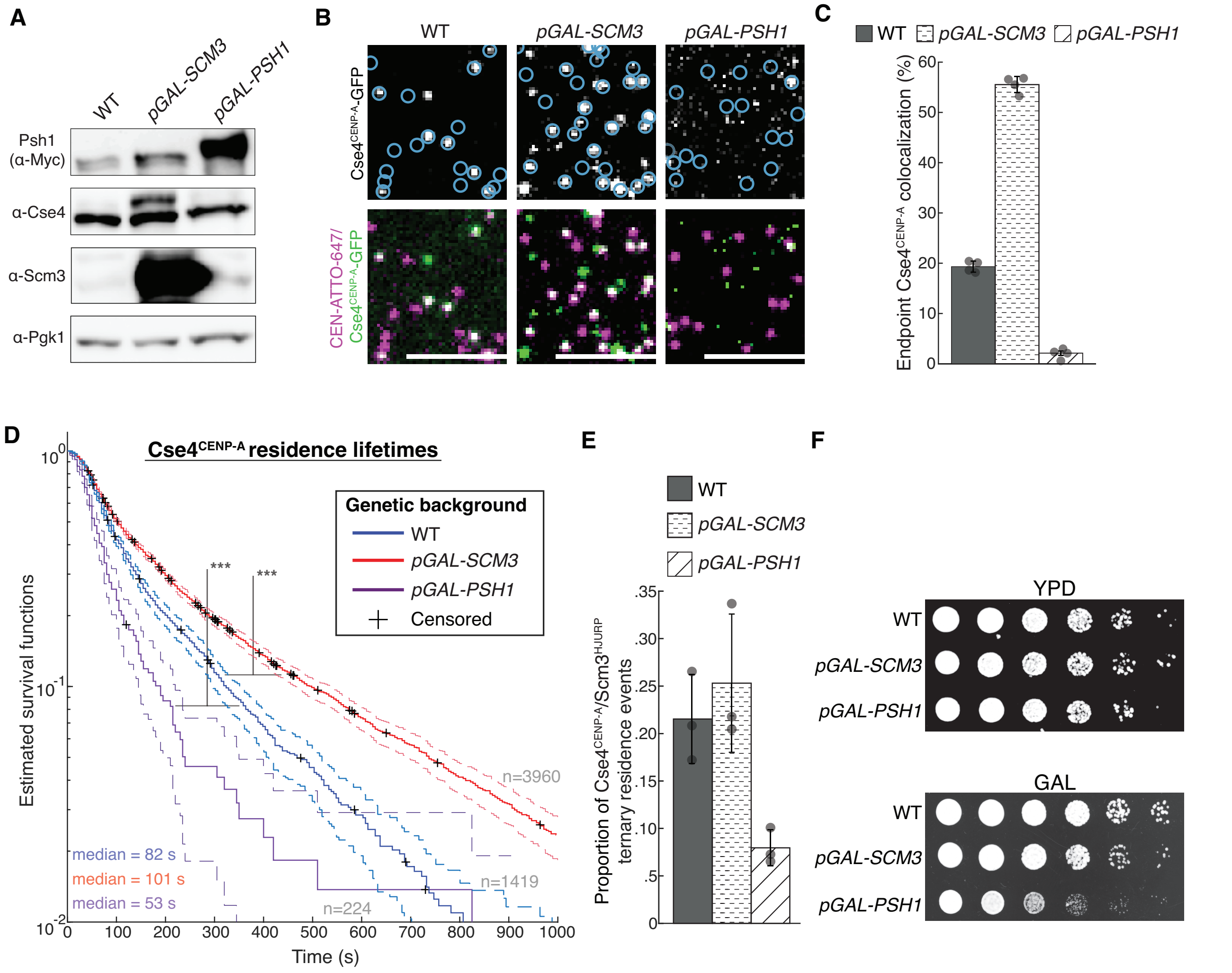

bioRxiv preprint doi: https://doi.org/10.1101/2023.01.20.524981; this version posted May 17, 2023. The copyright holder for this preprint (which was not certified by peer review) is the author/funder, who has granted bioRxiv a license to display the preprint in perpetuity. It is made available under aCC-BY 4.0 International license.

Figure EV4. Scm3^{HJURP}-Cse4^{CENP-A} complex is limiting for stable centromeric association of Cse4^{CENP-A}.

A Immunoblot analysis of whole cell extracts from WT, *pGAL-SCM3* and *pGAL-PSH1* cells using indicated antibodies (all panels cropped from the same blot).

B Example images of TIRFM endpoint colocalization assays. Top panels show visualized Cse4^{CENP-A} -GFP on CEN DNA in extracts from a WT genetic background (top-left panel) or extracts containing overexpressed Scm3^{HJURP} (*pGAL-SCM3,* top-middle panel) or overexpressed Psh1 (*pGAL-PSH1,* top-right panel) with colocalization shown in relation to identified CEN DNAs in blue circles. Bottom panels show overlay of DNA channel (magenta) with Cse4^{CENP-A} -GFP (green).

C Quantification of endpoint colocalization of Cse4^{CENP-A} on CEN DNA in extracts from a WT genetic background, extracts that contain overexpressed Scm3^{HJURP} or extracts that contain overexpressed Psh1 (19 $\pm$ 1.1%, 56 $\pm$ 1.6% and 2.1 $\pm$ 0.4% respectively, avg $\pm$ s.d. n=4 experiments, each examining ~1,000 DNA molecules from different extracts). Scale bars 3 μm.

D Estimated survival function plots of Kaplan-Meier analysis of residence lifetimes of Cse4^{CENP-A} on CEN DNA in extracts from WT genetic background (blue - median lifetime of 82 s, n=1419 over 3 experiments of ~1000 DNA molecules using different extracts), or from extracts that contain overexpressed Scm3^{HJURP} (red – median lifetime of 101 sec, n=3960 over 3 experiments of ~1000 DNA molecules using different extracts) or extracts that contain overexpressed Psh1 (purple – median lifetime of 52 sec, n=224 over 3 experiments of ~1000 DNA molecules using different extracts). Significant difference (***) between survival plots in WT extracts compared to those overexpressing Psh1 (two-tailed p-value of 8.9e-11 as determined by log-rank test) or Scm3^{HJURP} (two-tailed p-value of 0 as determined by log-rank test). 95% confidence intervals indicated (dashed lines), right-censored lifetimes (plus icons) were included and unweighted in survival function estimates.

bioRxiv preprint doi: https://doi.org/10.1101/2023.01.20.524981; this version posted May 17, 2023. The copyright holder for this preprint (which was not certified by peer review) is the author/funder, who has granted bioRxiv a license to display the preprint in perpetuity. It is made available under aCC-BY 4.0 International license.

E Proportion of ternary residences of Cse4^{CENP-A} with Scm3^{HJURP} on CEN DNA for WT extracts (0.22 ± .05, avg ± s.d. n=1419 over 3 experiments of ~1000 DNA molecules using different extracts), extracts containing overexpressed Scm3^{HJURP} (0.25 ± .07, avg ± s.d. n=3960 over 3 experiments of ~1000 DNA molecules using different extracts) and extracts containing overexpressed Psh1 (0.08 ± .02, avg ± s.d. n=224 over 3 experiments of ~1000 DNA molecules using different extracts). (**F**) Serial 5-fold dilutions of the following yeast strains were plated and grown two days on YPD and three days on galactose (GAL) at 23° C: WT (SBY21441), *pGAL-SCM3* (SBY21443), and *pGAL-PSH1* (SBY20836).

bioRxiv preprint doi: https://doi.org/10.1101/2023.01.20.524981; this version posted May 17, 2023. The copyright holder for this preprint (which was not certified by peer review) is the author/funder, who has granted bioRxiv a license to display the preprint in perpetuity. It is made available under aCC-BY 4.0 International license.

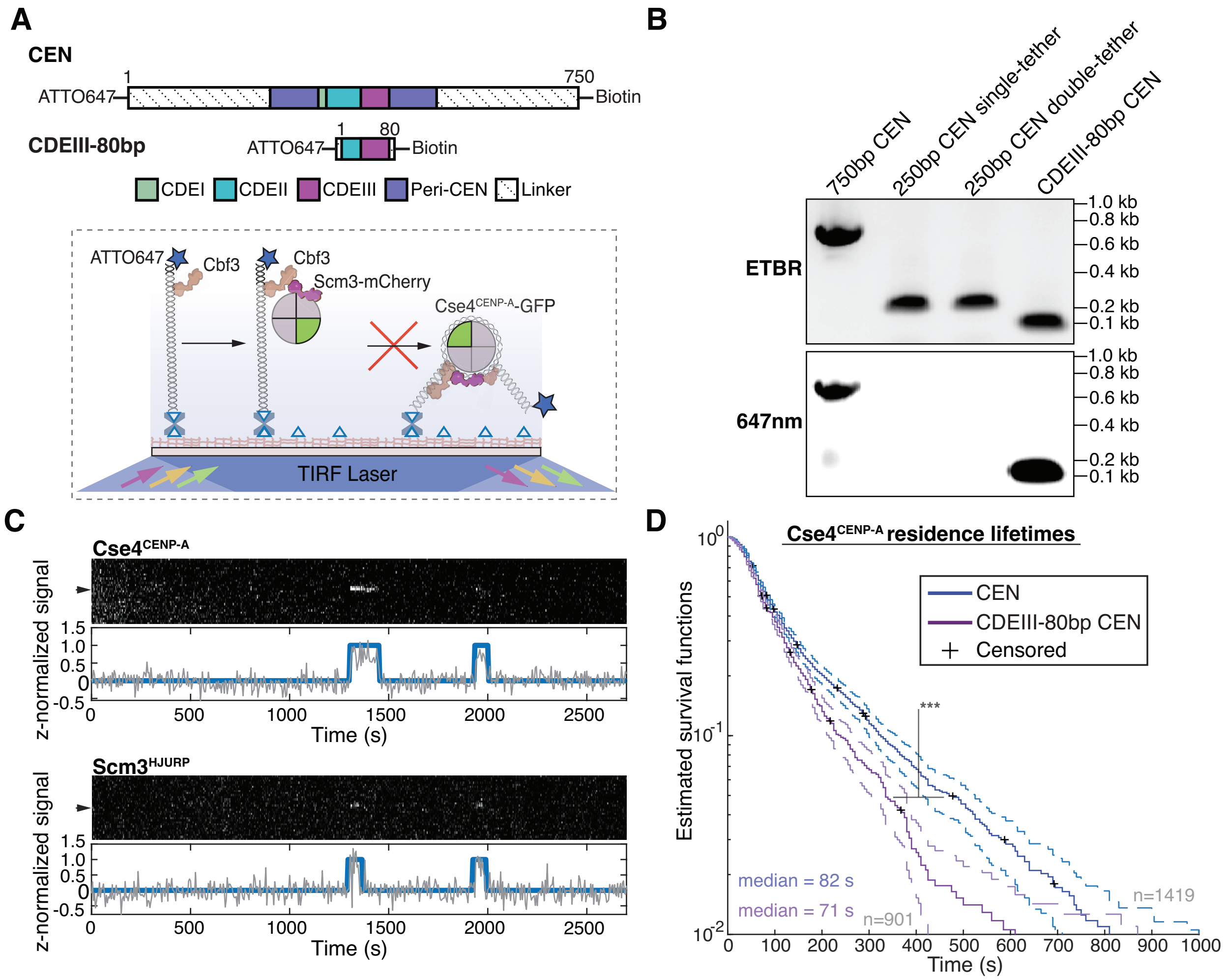

bioRxiv preprint doi: https://doi.org/10.1101/2023.01.20.524981; this version posted May 17, 2023. The copyright holder for this preprint (which was not certified by peer review) is the author/funder, who has granted bioRxiv a license to display the preprint in perpetuity. It is made available under aCC-BY 4.0 International license.

Figure EV5. Cse4^{CENP-A} residence lifetimes are significantly reduced on CDEIII-80 bp mutant CEN DNA.

A Schematic of overview of CDEIII-80 bp mutant CEN DNA, the canonical CEN DNA is shortened to 80 bp to prevent nucleosome formation and then similarly functionalized to the coverslip via a single biotin at the 5'end and functionalized with an organic dye at the free 3' end.

B CEN assembly templates including WT 750 bp CEN DNA, 250 bp single-tether CEN DNA, 250 bp double-tethered CEN DNA and CDEIII-80 bp CEN DNA as visualized via EtBr (top panel) or 647nm excitation (bottom panel) on a 1% agarose gel.

C Representative colocalization traces of Cse4^{CENP-A} and Scm3^{HJURP} on a single CDEIII-80 bp CEN DNA. Top panel includes kymograph of Cse4^{CENP-A} (top-488 nm) in relation to single identified CEN DNA (arrow), with normalized intensity trace (grey-bottom) as well as identified colocalization pulses (blue). Bottom panel includes kymograph of Scm3^{HJURP} (bottom-568 nm) in relation to the same identified CEN DNA (arrow), with normalized intensity trace (grey-bottom) as well as identified colocalization pulse (blue). Cases where identified pulses in Scm3^{HJURP} and Cse4^{CENP-A} coincide represent observed colocalization of both proteins on single CDEIII-80 bp CEN DNA. Images acquired every 5 seconds with normalized fluorescence intensity shown in arbitrary units.

D Estimated survival function plots of Kaplan-Meier analysis of all identified CEN DNA colocalization events of Cse4^{CENP-A} (blue - median lifetime of 82 s, n=1619 over 3 experiments of ~1000 DNA molecules using different extracts) and identified colocalization events on CDEIII-80 bp CEN DNA of Cse4^{CENP-A} (red – median lifetime of 71 s, n=901 over 3 experiments of ~1000 DNA molecules using different extracts). Significant difference (***) between CEN DNA and CDEIII-80 bp CEN DNA lifetime survival plots (two-tailed p-value of 1.0e-6 as determined by log-rank test). 95% confidence intervals indicated (dashed lines), right-censored lifetimes (plus icons) were included and unweighted in survival function estimates.
